# A Comparison of the “Reduced Losses” and “Increased Production” Models for Mussel Bed Dynamics

**DOI:** 10.1007/s11538-021-00932-1

**Published:** 2021-08-24

**Authors:** Jonathan A. Sherratt, Quan-Xing Liu, Johan van de Koppel

**Affiliations:** 1 Department of Mathematics and Maxwell Institute for Mathematical Sciences, Heriot-Watt University, Edinburgh, EH14 4AS UK; 2 State Key Laboratory of Estuarine and Coastal Research, School of Ecological and Environmental Sciences, East China Normal University, Shanghai, 200241 People’s Republic of China; 3 Department of Estuarine and Delta Systems, Royal Netherlands Institute for Sea Research and Utrecht University, PO Box 140, 4400 AC Yerseke, The Netherlands

**Keywords:** Pattern formation, Mathematical model, Mussels, Reaction–diffusion–advection

## Abstract

Self-organised regular pattern formation is one of the foremost examples of the development of complexity in ecosystems. Despite the wide array of mechanistic models that have been proposed to understand pattern formation, there is limited general understanding of the feedback processes causing pattern formation in ecosystems, and how these affect ecosystem patterning and functioning. Here we propose a generalised model for pattern formation that integrates two types of within-patch feedback: amplification of growth and reduction of losses. Both of these mechanisms have been proposed as causing pattern formation in mussel beds in intertidal regions, where dense clusters of mussels form, separated by regions of bare sediment. We investigate how a relative change from one feedback to the other affects the stability of uniform steady states and the existence of spatial patterns. We conclude that there are important differences between the patterns generated by the two mechanisms, concerning both biomass distribution in the patterns and the resilience of the ecosystems to disturbances.

## Introduction

Pattern formation at the landscape scale is an established feature of many ecosystems across the world. Examples include alternating patches of vegetation and bare ground in arid environments (Bastiaansen et al. 2018; Gandhi et al. 2019); “fairy circles” in Namibian grasslands (Zelnik et al. 2015); patterns of open-water pools in peatlands (Belyea 2007; Eppinga et al. 2009); tussock patterns in freshwater marshes (van de Koppel and Crain 2006; Yu 2010); and labyrinthine patterns in mussel beds (van de Koppel et al. 2005, 2008; Liu et al. 2014). Mathematical modelling has played an important role in the study of this type of pattern formation. Most commonly, models have been used to show that a hypothesised ecological mechanism can indeed generate spatial patterns. A crucial commonality for these underlying processes is that there is a local positive feedback, where organisms improve the conditions in which they grow, but at the same time lower growth conditions at distance, often by means of competition for resources. This scale-dependent alternation between local positive feedback and larger-scale negative feedback has been proposed for many patterned ecosystems, providing a general principle for regular spatial patterns for ecology and beyond. Despite this common ground, a wide variety of specific mechanisms have been proposed as potential generators of regular patterns. Soft-bottomed mussel beds provided an example of a system in which multiple hypothesised mechanisms have been shown to be potential generators of patterns. These large aggregations of mussels form in intertidal regions, most notably in the Dutch Wadden Sea, and are often patterned, with dense clusters of mussels separated by regions of bare sediment. Two possible mechanisms for this patterning have been proposed: (i) “reduced losses”—high mussel density reduces the dislodgement and predation of mussels, because of their adherence to one another; (ii) “increased production”—a high density of mussels increases their growth rate, since the faeces and pseudofaeces they produce raise them towards the algae-rich upper water layers. Mathematical modelling confirms that both of these mechanisms can generate spatial patterns (van de Koppel et al. 2005; Liu et al. 2012). How then can one determine which mechanism is the real driver of the observed pattern formation?

A first step in answering this question was taken by Liu and co-workers (Liu et al. 2012) who calculated bifurcation diagrams for models based on each of the two mechanisms, demonstrating significant differences between the patterns in the two models. However, it is unclear to what extent these differences depend on the formulation of the models and the parameter values, rather than being due to the alternative underlying patterning mechanisms. The objective of this paper is to address this issue in a comprehensive way. We will do this by developing a hybrid model that includes both the “reduced losses” and “increased production” mechanisms, with their relative importance controlled by a single tuning parameter. Our hybrid model is based on the “reduced losses” model proposed by van de Koppel et al. (2005) and the “increased production” model proposed by Liu et al. (2012).

We begin by describing the two models separately, starting with the “reduced losses” model (RLM). Denoting the mussel density by $\widetilde{m}(\widetilde{x},\widetilde{t}\,)$ and the density of algae (the main mussel food source Dolmer 2000; Øie et al. 2002) by $\widetilde{a}(\widetilde{x},\widetilde{t}\,)$, the model equations are: 1a1b Here $\widetilde{t}$ is time and $\widetilde{x}$ is distance away from the shore; $\widetilde{A}$, $\widetilde{B}$, $\widetilde{C}$, $\widetilde{D}$, $\widetilde{E}$, $\widetilde{F}$, $\widetilde{K}$ and $\widetilde{V}$ are positive parameters. Since its initial formulation in 2005 (van de Koppel et al. 2005), model () and minor extensions have been studied in a number of papers, from both applications and mathematical viewpoints (Wang et al. 2009; Liu et al. 2012; Sherratt 2013; Ghazaryan and Manukian 2015; Cangelosi et al. 2014; Sherratt and Mackenzie 2016).

The alternative “increased production” model (IPM) centres around the build-up of faeces and pseudofaeces (van Broekhoven et al. 2015) under mussel beds, which contribute to the underlying sediment. Since the mussels’ food source (algae) mainly resides in upper water layer, this sediment deposition raises the mussels towards their food source and thus further promotes their growth (Liu et al. 2012, 2014). In this case, we denote the mussel and algal densities by $\widehat{m}(\widehat{x},\widehat{t}\,)$ and $\widehat{a}(\widehat{x},\widehat{t}\,)$, respectively, and we use $\widehat{s}(\widehat{x},\widehat{t}\,)$ to represent the amount of accumulated sediment. Here $\widehat{t}$ and $\widehat{x}$ are time and distance away from the shore, respectively. Then, the model of Liu et al. (2012) is: 2a2b2c where $\widehat{A}$, $\widehat{B}$, $\widehat{\eta }$ ($<1$), $\widehat{S}_0$, $\widehat{C}$, $\widehat{D}_m$, $\widehat{D}_s$, $\widehat{E}$, $\widehat{F}$, $\widehat{P}$, $\widehat{Q}$ and $\widehat{V}$ are positive parameters. The function $\bigl (\widehat{s}+\widehat{\eta }\widehat{S}_0\bigr )\big / \bigl (\widehat{s}+\widehat{S}_0\bigr )$ has the property of increasing from a nonzero value ($\widehat{\eta }$) when $\widehat{s}=0$ towards the saturation level 1 as $\widehat{s}\rightarrow \infty$; this reflects the increase in the growth rate of the mussel population with increased proximity to the (algal-rich) upper water layers. Beyond these properties, the precise functional form is arbitrary.

Liu et al. (2012) calculated bifurcation diagrams for models () and () as a way of comparing their predictions for pattern formation. One of their observations was a distinct difference between the mussel densities in the patterns predicted by the two models. They reported that the average mussel density within patterns in the reduced losses model () is considerably greater than that in the spatially uniform steady state, but that in the increased production model () the two densities are similar. In this paper, we will present a more detailed study of this difference between the models with the aim of clarifying whether it is due to different model formulations and parameters, or whether it can genuinely be attributed to the different underling mechanisms. The difference is important because it provides a potential avenue for testing which model applies in a particular ecological context; mere observation of patterns is not sufficient for this since both models predict pattern formation for a wide range of ecologically plausible parameters. One issue that necessitates a detailed study is that (as we will show) there are multiple patterned solutions for any given set of ecological parameters.

**Fig. 1:**
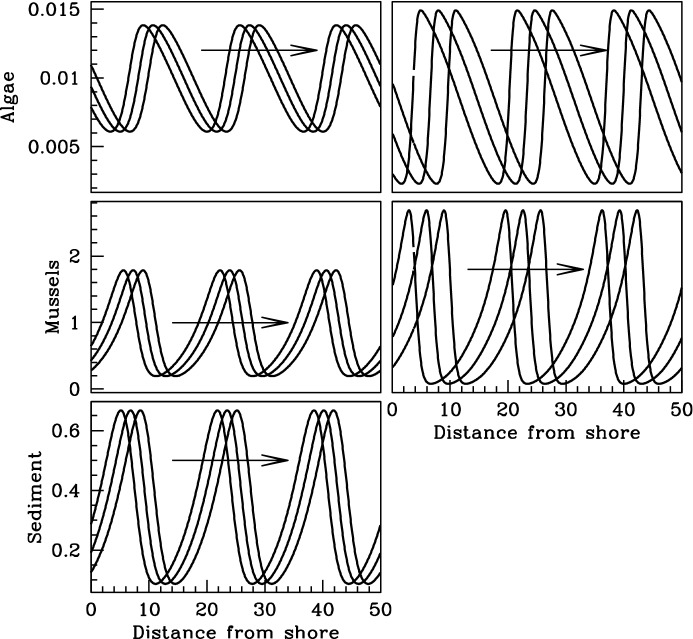
A comparison of pattern solutions of the full increased production model () and the reduced model (). Before solving, we nondimensionalised the models. Rescalings (6) are not convenient for (), and so we used a different set of rescalings taken from Liu et al. (2012); details are given in “Appendix”. This transforms () and () to ( ) and (), respectively. We used the domain $0<\check{x}<50$ with periodic boundary conditions, and with parameter values $\check{\alpha }=50$, $\check{\beta }=200$, $\check{\eta }=0.1$, $\check{\nu }=360$, $\check{D}=1$, $\check{\delta }=320$, $\check{\theta }=2.5$ and $\check{\delta }=1$. (All notations are defined in “Appendix”). We began by solving (), with the variables $(\check{a},\check{m},\check{s})$ set to the spatially uniform steady state $(\check{a}_s,\check{m}_s,\check{s}_s)$ plus the small mixed-mode perturbation $0.01(\check{a}_s,\check{m}_s,\check{s}_s)\sum _{i=1}^{i=10}\cos (\pi \,i\,\check{x}/50)$. The solutions were plotted at times $\check{t}=600,\,602,\,604$. We then used the solutions for $\check{a}$ and $\check{m}$ at $\check{t}=604$ as initial conditions for (), again solving from $\check{t}=0$ and plotting the solutions at times $\check{t}=600,\,602,\,604$, although we translated the solutions in the negative $\check{x}$ direction by 3.75 space units in order to facilitate comparison. (Since we are using periodic boundary conditions, the solutions are invariant to translation.)

As a first step, we consider a two-equation analogue of (), which will facilitate direct comparison between the two model frameworks. Our aim is not to obtain a formal two-equation approximation to (); rather, we seek a prototypical model of the increased production mechanism, and we use () as a starting point for formulating this. In this spirit, we will apply two simplifications to (), neither of which is a good quantitative approximation, but both of which maintain the key qualitative features of the model. Firstly, we make a quasi-steady-state assumption on the kinetics of $\widehat{s}$, setting $\widehat{s}=\bigl (\widehat{P}\big /\widehat{Q}\bigr )\,\widehat{m}$ in (2a,b). In order for this to be a good quantitative approximation, it would be necessary that $\widehat{P}$ and $\widehat{Q}$ are significantly larger than comparable rate parameters in other equations. This is not actually the case: the timescale of sediment production and erosion is broadly similar to that of mussel birth and death, and for example the values estimated by Liu et al. (2012) for $\widehat{P}$ and $\widehat{Q}$ are the same. However, $\widehat{s}$ does not play a central role in the pattern formation process, and the only effects of the quasi-steady-state assumption are on quantitative details. Secondly, to simplify the two-equation model we replace $\bigl (\widehat{s}+\widehat{\eta }\widehat{S}_0\bigr )\big /\bigl (\widehat{s}+\widehat{S}_0\bigr )$ by $\widehat{s}\big /\widehat{S}_0$. Quantitative validity of this approximation would require that $\widehat{S}_0\gg \widehat{s}\gg \widehat{\eta }\widehat{S}_0$, which holds for many of the patterns predicted by () using the parameter estimates in Liu et al. (2012), but not all. Note that (Liu et al. 2012) does not actually show any plots of the sediment profile in the patterned solutions of (); we show an example in Fig. 1. In fact, the choice of functional form for the dependence of mussel birth on sediment level $\widehat{s}$ in Liu et al. (2012) was somewhat arbitrary, and the prediction of pattern formation is not sensitive to this functional form. In particular, the nonzero value of $\bigl (\widehat{s}+\widehat{\eta }\widehat{S}_0\bigr )\big /\bigl (\widehat{s}+\widehat{S}_0\bigr )$ when $\widehat{s}=0$ is not important, for the following reason. In Fig. 1, it is clear that the mussel and sediment patterns are approximately in phase; this is a typical feature of simulations, and it has a mathematical basis from linear stability analysis that we outline in “Appendix”. It follows that if $\widehat{s}$ is small then $\widehat{m}$ will also be small, implying that the growth rate will necessarily be small.

These two simplifications give the model

3a $$\begin{aligned} \partial \widehat{a}/\partial \widehat{t}= & {} \widehat{F}\cdot \bigl (\widehat{A}-\widehat{a}\bigr )- \bigl (\widehat{B}\,\widehat{P}\big /\widehat{Q}\widehat{S}_0\bigr )\,\widehat{a}\,\widehat{m}^{2} +\widehat{V}\,\partial \widehat{a}/\partial \widehat{x} \end{aligned}$$

3b $$\begin{aligned} \partial \widehat{m}/\partial \widehat{t}= & {} \bigl (\widehat{C}\,\widehat{P}\big /\widehat{Q}\widehat{S}_0\bigr )\,\widehat{a}\,\widehat{m}^{2} -\widehat{E}\,\widehat{m} +\widehat{D}_m\,\partial ^2\widehat{m}/\partial \widehat{x}^{2} . \end{aligned}$$

Numerical studies show that the qualitative features of pattern formation in () are broadly similar to those in (); an example comparison is shown in Fig. 1. However, this similarity is not a key foundation of our study. Rather, our approach is to regard () as a prototypical model for the “increased production” mechanism, which can easily compared to the prototypical model () for the “reduced losses” mechanism.

Our next step is to nondimensionalise the two two-equation models. Previous papers on () and () have reduced them to dimensionless forms (van de Koppel et al. 2005; Wang et al. 2009; Liu et al. 2012); however if one applies these rescalings to () and (), respectively, then the resulting pairs of equations are not easily comparable. We use different rescalings that are constructed specifically to facilitate comparison between the two dimensionless models. We give the rescalings in full because our subsequent work depends crucially on the way in which the (dimensional) algal supply rate affects the dimensionless parameters in the two models. For (), we substitute

4 $$\begin{aligned} \widetilde{a}^{*}= & {} \bigl (\widetilde{C}\big /\widetilde{F}\bigr )\widetilde{a} \qquad \widetilde{m}^{*}=\bigl (\widetilde{B}\big /\widetilde{F})\widetilde{m} \qquad \widetilde{x}^{*}=\bigl (\widetilde{F}\big /\widetilde{D}\bigr )^{1/2}\widetilde{x} \qquad \widetilde{t}^{*}=\widetilde{F}\,\widetilde{t} \nonumber \\ \widetilde{\alpha }^{*}= & {} \widetilde{A}\,\widetilde{C}\big /\widetilde{F} \qquad \widetilde{\beta }^{*}=\widetilde{E}\big /\bigl (\widetilde{F}\,\widetilde{K}\bigr ) \qquad \widetilde{\xi }^{*}=\widetilde{F}\big /\bigl (\widetilde{B}\,\widetilde{K}\bigr ) \qquad \widetilde{\nu }^{*}=\widetilde{V}\big /\bigl (\widetilde{D}^{1/2}\widetilde{F}^{1/2}\bigr )\nonumber \\ \end{aligned}$$

which gives

5a $$\begin{aligned} \partial \widetilde{a}^{*}/\partial \widetilde{t}^{*}= & {} \widetilde{\alpha }^{*}-\widetilde{a}^{*}-\widetilde{a}^{*}\widetilde{m}^{*} +\widetilde{\nu }^{*}\,\partial \widetilde{a}^{*}/\partial \widetilde{x}^{*} \end{aligned}$$

5b $$\begin{aligned} \partial \widetilde{m}^{*}/\partial \widetilde{t}^{*}= & {} \widetilde{a}^{*}\widetilde{m}^{*}-\widetilde{\beta }^{*}\widetilde{m}^{*}\big /\bigl (1+\widetilde{\xi }^{*}\widetilde{m}^{*}\bigr ) +\partial ^2\widetilde{m}^{*}/\partial \widetilde{x}^{*\,2} . \end{aligned}$$

Here the asterisks denote dimensionless variables and parameters. The dimensionless parameters $\widetilde{\alpha }^{*}$, $\widetilde{\nu }^{*}$, $\widetilde{\xi }^{*}$ and $\widetilde{\beta }^{*}$ are defined by a combination of dimensional constants, but readers may find it useful to interpret intuitively $\widetilde{\alpha }^{*}$ as corresponding to the algal supply from upper water layers, $\widetilde{\nu }^{*}$ as corresponding to the tide strength, $\widetilde{\xi }^{*}$ as corresponding to the ability of intermussel bonds to reduce dislodgement by waves, and $\widetilde{\beta }^{*}$ as corresponding to the per capita dislodgement rate for isolated mussels.

For (), we substitute

6 $$\begin{aligned} \widehat{a}^{*}= & {} \frac{\widehat{C}\widehat{P}^{1/2}}{\widehat{B}^{1/2}\widehat{F}^{1/2}\widehat{Q}^{1/2}\widehat{S}_0^{1/2}}\,\widehat{a} \qquad \! \widehat{m}^{*}=\left( \frac{\widehat{B}\widehat{P}}{\widehat{F}\widehat{Q}\widehat{S}_0}\right) ^{\!1/2}\!\widehat{m} \qquad \! \nonumber \\ \widehat{x}^{*}= & {} \bigl (\widehat{F}/\widehat{D}_m\bigr )^{1/2} \widehat{x} \qquad \! \widehat{t}^{*}=\widehat{F}\,\widehat{t} \nonumber \\ \widehat{\alpha }= & {} \frac{\widehat{A}\widehat{C}\widehat{P}^{1/2}}{\widehat{B}^{1/2}\widehat{F}^{1/2}\widehat{Q}^{1/2}\widehat{S}_0^{1/2}} \qquad \widehat{\beta }=\widehat{E}/\widehat{F} \qquad \widehat{\nu }=\widehat{V}\big /\bigl (\widehat{D}_m\widehat{F}\bigr )^{1/2} \end{aligned}$$

which gives

7a $$\begin{aligned} \partial \widehat{a}^{*}/\partial \widehat{t}^{*}= & {} \widehat{\alpha }^{*}-\widehat{a}^{*}-\widehat{a}^{*}\widehat{m}^{*\,2} +\widehat{\nu }^{*}\,\partial \widehat{a}^{*}/\partial \widehat{x}^{*} \end{aligned}$$

7b $$\begin{aligned} \partial \widehat{m}^{*}/\partial \widehat{t}^{*}= & {} \widehat{a}^{*}\widehat{m}^{*\,2}-\widehat{\beta }^{*}\widehat{m}^{*} +\partial ^2\widehat{m}^{*}/\partial \widehat{x}^{*\,2} . \end{aligned}$$

Again the asterisks denote dimensionless variables and parameters. Equations () are the Klausmeier model, which has been well studied as a model of semi-arid vegetation (Klausmeier 1999; Sherratt and Lord 2007; van der Stelt et al. 2013), and whose kinetics are the same as those of the Gray–Scott model, which has important applications to chemistry (Chen and Ward 2009; Doelman et al. 1997; Gray and Scott 1984). The dimensionless parameters $\widehat{\alpha }$, $\widehat{\nu }$ and $\widehat{\beta }$ are defined by a combination of dimensional constants, but readers may find it useful to interpret intuitively $\widehat{\alpha }$ as corresponding to the algal supply from upper water layers, $\widehat{\nu }$ as corresponding to the tide strength, and $\widehat{\beta }$ as corresponding to the per capita dislodgement rate of mussels by waves.

The basic approach of this paper is to construct a hybrid model, which includes both the reduced losses and increased production feedback mechanisms. The model will include a new (dimensionless) parameter $\lambda$, which takes values between 0 and 1 and which controls the relative importance of the two mechanisms, with the equations reducing to () when $\lambda =1$ and to () when $\lambda =0$. This approach is made possible by the close similarity between the two pairs of dimensionless equations () and (), and our hybrid model is:

8a $$\begin{aligned} \partial {a}/\partial {t}= & {} {\alpha }-{a}-{a}{m}\left[ \lambda +(1-\lambda ){m}\right] +{\nu }\,\partial {a}/\partial {x} \end{aligned}$$

8b $$\begin{aligned} \partial {m}/\partial {t}= & {} {a}{m}\left[ \lambda +(1-\lambda ){m}\right] -{\beta }{m}\left[ 1-\lambda +\lambda \big /\bigl (1+{\xi }{m}\bigr ) \right] +\partial ^2{m}/\partial {x}^2 . \end{aligned}$$

Note that a dimensional model can be recovered from () by using either (4) or (6) as rescalings.

**Fig. 2:**
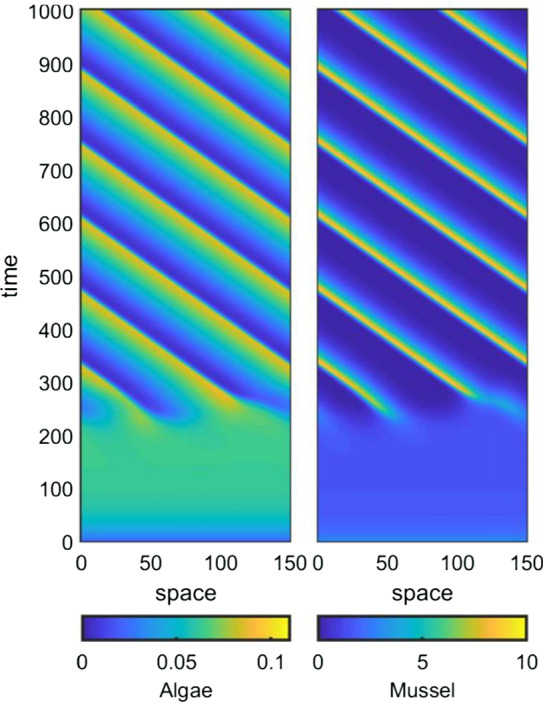
A typical pattern solution of the hybrid model (). We show the evolution of a pattern following a small random perturbation of a uniform state. The parameter values are $\alpha =0.2$, $\beta =0.1$, $\nu =100$, $\xi =0.5$, $\lambda =0.25$

Figure 2 shows a typical example of a spatially patterned solution of (). A central question that we will address in this paper is how the mussel density in such patterns changes as the supply rate of algae is varied. In view of this, it is important to note that the algal supply parameters $\widetilde{A}$ in () and $\widehat{A}$ in () do not appear in the rescalings for $\widetilde{m}^{*}$ or $\widehat{m}^{*}$ and that the only dimensionless parameters that they affect are $\widetilde{\alpha }^{*}$ and $\widehat{\alpha }^{*}$. Therefore, we can address our question by considering how the (dimensionless) mussel density *m* in patterned solutions of () changes with the (dimensionless) parameter $\alpha$.

## Uniform Steady States of the Hybrid Model

For all parameter values, model () has a mussel-free steady state $a=\alpha$, $m=0$. Spatially uniform steady states with $m\not =0$ must satisfy

9 $$\begin{aligned} a= & {} \alpha \big /\bigl [1+\lambda {}m+(1-\lambda )m^2\bigr ] \end{aligned}$$

10 $$\begin{aligned} 0= & {} \alpha \left[ \lambda +(1-\lambda )m\right] \cdot (1+\xi m) -\beta \left[ \lambda +(1-\lambda )\xi {}m\right] \cdot \left[ 1+\lambda {}m+(1-\lambda )m^2\right] .\nonumber \\ \end{aligned}$$

Equation (10) is a cubic polynomial whose roots are very complicated algebraically, and we investigated them using numerical continuation. Starting points for this are provided by the extreme cases $\lambda =0$ (increased production model) and $\lambda =1$ (reduced losses model), for which the spatially uniform steady states have a much simpler form; moreover, their stability can be determined quite easily; here and throughout this section, the “stability” that we consider is to spatially uniform perturbations. For $\lambda =0$, $(\alpha ,0)$ is stable for all parameter values, and if $\alpha >2\beta$ there are two other steady states:

11 $$\begin{aligned} a=\frac{2\beta ^2}{\alpha -\sqrt{\alpha ^2-4\beta ^2}} m=\frac{\alpha -\sqrt{\alpha ^2-4\beta ^2}}{2\beta } \end{aligned}$$

which is unstable and

12 $$\begin{aligned} a=\frac{2\beta ^2}{\alpha +\sqrt{\alpha ^2-4\beta ^2}} m=\frac{\alpha +\sqrt{\alpha ^2-4\beta ^2}}{2\beta }. \end{aligned}$$

The stability of (12) depends on parameters, but it is always stable if $\beta <2$ which holds for realistic parameter estimates (Liu et al. 2012). For $\lambda =1$, $(\alpha ,0)$ is stable if and only if $\alpha <\beta$. When $(\alpha /\beta )$ lies between 1 and $(1/\xi )$, there is one other nonnegative steady state

13 $$\begin{aligned} a=(\beta -\xi \alpha )/(1-\xi )m=(\alpha -\beta )/(\beta -\xi \alpha ) \end{aligned}$$

which is stable if $\xi <1$.

**Fig. 3:**
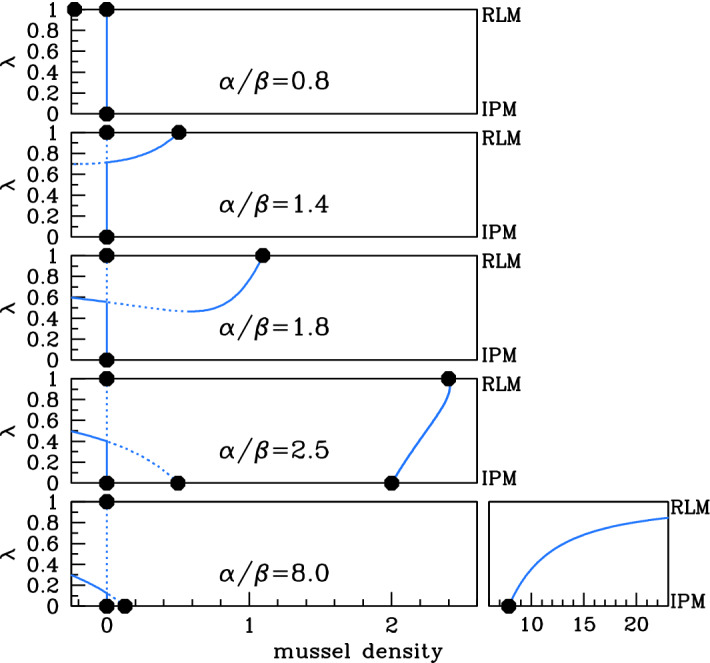
Steady states for $\xi <1$. We plot the steady-state solutions of () when $\xi =0.15$, which is typical of behaviour when $\xi <1$. We plot mussel density *m* against the parameter $\lambda$. Solid $\big /$ dashed lines indicate stable $\big /$ unstable steady states, calculated by numerical continuation using auto (Doedel 1981; Doedel et al. 1991, 2006), with $\lambda$ as continuation parameter. Dots indicate the steady states for the reduced losses ($\lambda =1$) and increased production ($\lambda =0$) models; these are $(a,m)=(\alpha ,0)$ plus (11), (12) and (13). The five different cases shown in the figure are separated by four critical values of $\alpha /\beta$: 1, $1/\lambda _{\text {{}min}}^{0}\approx 1.55$, 2, and $1/\xi \approx 6.67$; a full description is given in the main text. To improve clarity, the plot for $\alpha /\beta =8.0$ is shown in two parts with different horizontal scalings. The stability of the steady states shown in the figure is valid, provided that $\beta <2$, which applies for realistic parameter estimates (Liu et al. 2012)

**Fig. 4:**
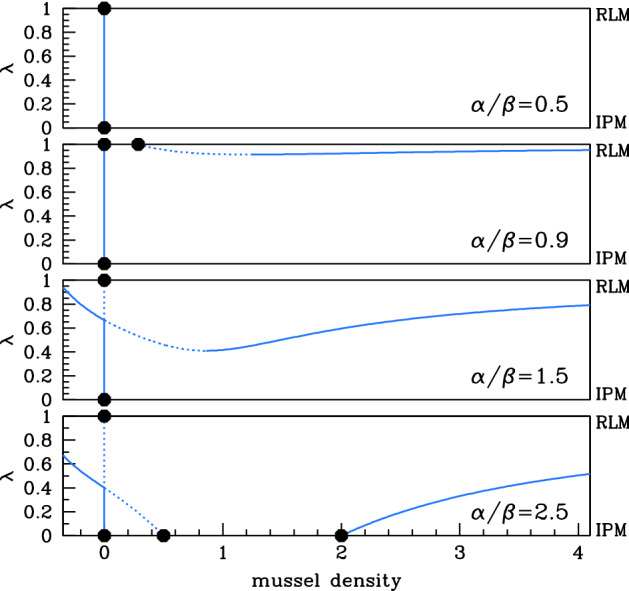
Steady states for $\xi >1$. We plot the steady-state solutions of () when $\xi =1.5$, which is typical of behaviour when $\xi >1$. We plot mussel density *m* against the parameter $\lambda$. Solid $\big /$ dashed lines indicate stable $\big /$ unstable steady states, calculated by numerical continuation using auto (Doedel 1981; Doedel et al. 1991, 2006), with $\lambda$ as the continuation parameter. Dots indicate the steady states for the reduced losses ($\lambda =1$) and increased production ($\lambda =0$) models; these are $(a,m)=(\alpha ,0)$ plus (11), (12) and (13). The four different cases shown in the figure are separated by three critical values of $\alpha /\beta$: $1/\xi \approx 0.67$, 1 and 2; a full description is given in the main text. The steady-state solution branches depend on $\alpha$ and $\beta$ only through their ratio $\alpha /\beta$; their stability does depend on the separate values, but the stability of the steady states shown in the figure is valid whenever $0<\beta <2$, which applies for realistic parameter estimates (Liu et al. 2012). Figure 7 shows the corresponding bifurcation diagram for a larger value of $\beta$

**Fig. 5:**
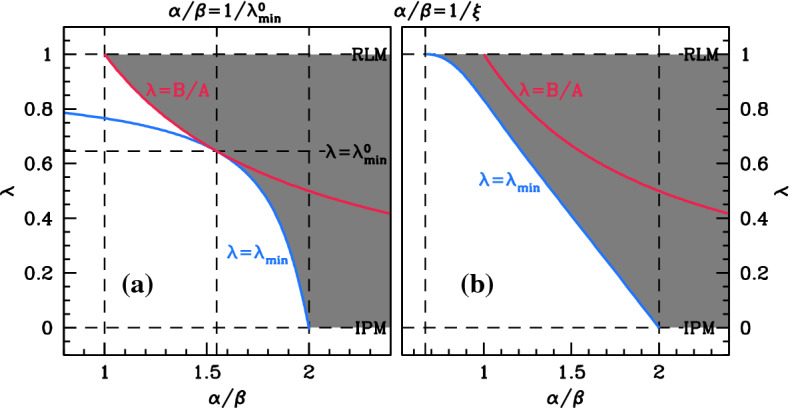
Existence of a positive stable steady state. The shading shows typical examples of the parameter region for which model () has a positive steady state that is stable to homogeneous perturbations, for **a** $\xi <1$ and **b** $\xi >1$. The blue curve is the locus of $\lambda _{\text {{}min}}$, calculated by a two-parameter numerical continuation using auto (Doedel 1981; Doedel et al. 1991, 2006), with $\lambda$ and $\alpha$ as continuation parameters. The red curve is $\alpha /\beta =1/\lambda$: the mussel-free steady state $a=\alpha$, $m=0$ is stable below this curve and unstable above it. The parameter values used for the plots were $\beta =0.1$ and **a** $\xi =0.15$, **b** $\xi =1.5$

With these expressions for the steady states when $\lambda =0$ and $\lambda =1$ as starting points, we used the software package auto (Doedel 1981; Doedel et al. 1991, 2006) to track the steady states and their stability as $\lambda$ is varied. The results are significantly different when $\xi$ is above and below 1, and Figs. 3 and 4 illustrate the two cases. We will discuss first the case $\xi <1$ (Fig. 3) and then $\xi >1$ (Fig. 4), before using our results to construct a diagram (Fig. 5) showing the existence of a positive stable-steady state in a parameter plane. However, a few general comments about the stability of the steady states are a useful preliminary. The form of (10) shows that the steady states depend on the parameters $\alpha$ and $\beta$ only through the ratio $\alpha /\beta$. In general, their stability does depend on the individual values of $\alpha$ and $\beta$, but we will show later that when $\beta <2$, the stability also depends only on the ratio $\alpha /\beta$. Realistic parameter estimates are consistent with $\beta <2$ (Liu et al. 2012), and therefore, the stability shown in Figs. 3 and 4 applies under this assumption. In the figures, we plot the mussel density *m* at the steady states against $\lambda$, for a series of values of the parameter ratio $\alpha /\beta$. Recall that $\alpha$ and $\beta$ are most usefully interpreted as representing the rates of algal supply and mussel loss, respectively, although of course they are dimensionless parameter ratios that involve a combination of ecological quantities (see (4) and (6)).

Considering first $\xi <1$, five different cases are shown in Fig. 3. When $\alpha /\beta$ is small, the corresponding algal supply is insufficient to maintain a mussel population for any value of $\lambda$, and the only steady state is the mussel-free state, which is always stable. As $\alpha /\beta$ increases through 1, (13) becomes positive, and there is a stable steady state for sufficiently large values of $\lambda$. This solution branch actually has a local minimum, at $\lambda =\lambda _{\text {{}min}}$ say. The value of *m* giving this local minimum is negative when $\alpha /\beta$ is just above 1, but it increases with $\alpha /\beta$ and passes through zero when (10) has a double root at $m=0$. A straightforward calculation shows that this occurs when $\alpha /\beta =1/\lambda _{\text {{}min}}^{0}$, where $\lambda _{\text {{}min}}^{0}$ is the corresponding value of $\lambda$ and is given by

$$\begin{aligned} \lambda _{\text {{}min}}^{0}=\frac{\sqrt{5-4\xi }-1}{2(1-\xi )}. \end{aligned}$$

For the value of $\xi$ used in Fig. 3, $\lambda _{\text {{}min}}^{0}\approx 0.646\Rightarrow 1/\lambda _{\text {{}min}}^{0}\approx 1.55$. The numerical results indicate a change in stability at the local minimum, as one would expect from general bifurcation theory: $\lambda =\lambda _{\text {{}min}}$ is a saddle–node bifurcation point (Kuznetsov 2004, Ch. 3). Therefore, when $(\alpha /\beta )$ is just above $1/\lambda _{\text {{}min}}^{0}$, there are two positive steady states for $\lambda >\lambda _{\text {{}min}}$, one stable and one unstable. Figure 5a shows that $\lambda _{\text {{}min}}$ decreases as $(\alpha /\beta )$ increases, reaching 0 at $\alpha /\beta =2$. This heralds the appearance of nonzero steady states for $\lambda =0$ (the increased production model case): see (11) and (12). The final change in qualitative behaviour occurs when $\alpha /\beta =1/\xi$, when the value of *m* at the nonzero steady state (13) for $\lambda =1$ passes through infinity and becomes negative. For $\alpha /\beta >1/\xi$, the solution branch starting at (12) when $\lambda =0$ does not intersect $\lambda =1$; instead, $m\rightarrow \infty$ as $\lambda \rightarrow {}1^{-}$. This is illustrated in the right-hand subpanel of Fig. 3 for $\alpha /\beta =8$.

The onset of patterning in () occurs when a spatially uniform steady state becomes unstable to inhomogeneous perturbations—this is a Turing or Turing–Hopf bifurcation. A prerequisite for this is a positive steady state that is stable to homogeneous perturbations, and the above results enable us to determine when such a steady state exists (for $\xi <1$). Firstly, we note that there is never more than one positive stable steady state.^1^ When $\alpha /\beta >2$, there is a stable steady for all $\lambda$, with the exception of $\lambda =1$ when $\alpha /\beta >1/\xi$. For $2>\alpha /\beta >1/\lambda _{\text {{}min}}^{0}$, the condition for a positive steady state is $\lambda >\lambda _{\text {{}min}}$, while for $1/\lambda _{\text {{}min}}^{0}>\alpha /\beta >1$ the condition is $\lambda >(\beta /\alpha )$, since $m=0$ is a solution of (10) when $\alpha /\beta =1/\lambda$. Finally when $\alpha /\beta <1$, there are no positive stable steady states. Figure 5a illustrates the parameter region giving a positive stable steady state for one value of $\xi$ between 0 and 1.

We now consider the case of $\xi >1$, which is a little simpler (Fig. 4) . Again, when $\alpha /\beta$ is small, the only steady state is the mussel-free state, which is always stable. As $\alpha /\beta$ increases through $1/\xi$, a branch of positive steady states appears. From the viewpoint of bifurcation theory, this solution branch arises from a transcritical bifurcation at $(a,m)=(0,\infty )$ when $\lambda =1$; for fixed $\lambda <1$, the appearance of the steady states corresponds to a saddle–node bifurcation as $\alpha /\beta$ is increased. As for $\xi <1$, the solution branch has a local minimum, at $\lambda =\lambda _{\text {{}min}}$ say, which separates stable and unstable parts of the solution branch. At $\alpha /\beta =1$, the mussel density at the nontrivial steady state (13) for $\lambda =1$ changes sign, and for $\alpha /\beta >1$ the mussel-free steady state is unstable for values of $\lambda$ above the intersection point of the two solution branches. In the language of bifurcation theory, there is another transcritical bifurcation, this time at $(a,m)=(\alpha ,0)$ when $\lambda =1$. The value of $\lambda _{\text {{}min}}$ decreases as $\alpha /\beta$ increases, and it reaches zero at $\alpha /\beta =2$, heralding the appearance of positive steady states for $\lambda =0$. Figure 5b shows a typical plot of $\lambda _{\text {{}min}}$ against $\alpha /\beta$ for $\xi >1$. This curve bounds the parameter region giving a positive steady state, which is shaded in the figure. Note that for $\alpha /\beta >1$ there is a positive steady state for an interval of $\lambda$ values up to 1 (including $\lambda =0$), but not for $\lambda =1$ itself. Figure 5b illustrates the parameter region giving a positive stable steady state for one value of $\xi$ greater than 1.

To conclude our discussion of steady states, we return to the issue of their stability; specifically, we will justify our assertion that provided $\beta <2$, stability depends on the parameters $\alpha$ and $\beta$ only through their ratio $\alpha /\beta$. For a system of two coupled odes  the stability of a steady state depends on the trace and determinant of the Jacobian (a.k.a. stability or community) matrix. For a steady state of () with $m\not =0$, the determinant of the Jacobian matrix depends on $\alpha$ and $\beta$ only through their ratio $\alpha /\beta$, while the trace has the form ${{\mathcal {T}}}={{\mathcal {F}}}_1\beta -{{\mathcal {F}}}_2$. Here ${{\mathcal {F}}}_1$ and ${{\mathcal {F}}}_2$ are both strictly positive and depend on $\lambda$, $\xi$ and the ratio $\alpha /\beta$; their algebraic forms are complicated^2^. However, since ${{\mathcal {F}}}_2>0$ it follows that for any set of values of $\lambda$, $\xi$ and $\alpha /\beta$, ${{\mathcal {T}}}<0$ for sufficiently small $\beta$. Then, the steady state is stable $\big /$ unstable when the determinant of the Jacobian matrix is positive $\big /$ negative, which depends on $\alpha$ and $\beta$ only through their ratio $\alpha /\beta$. In the latter case, the steady state is unstable for all $\beta$, but when the determinant is positive, the steady state will only be stable for $\beta <\beta _{{{}{\mathrm{crit}}}}={{\mathcal {F}}}_2\big /{{\mathcal {F}}}_1$. Thus for $\beta >\beta _{{{}{\mathrm{crit}}}}$ there are no stable steady states with positive mussel density.

**Fig. 6:**
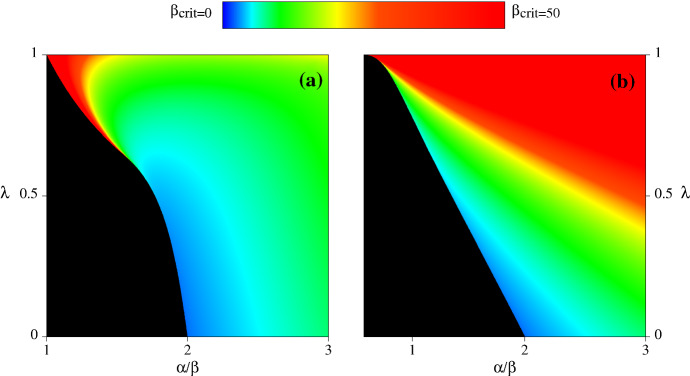
$\beta _{{{}{\mathrm{crit}}}}$ for (a) ${\xi }={0.15}$, (b) ${\xi }={1.5}$. For values of $\beta$ above $\beta _{{{}{\mathrm{crit}}}}$, there are no stable steady states with positive mussel density. The colour indicates $\beta _{{{}{\mathrm{crit}}}}$ according to the scalebar, with black indicating that there is no positive steady state. Thus, the black region in these plots corresponds to the unshaded region in Figs. 3 and 4. The key message from these plots is that in both cases the minimum value of $\beta _{{{}{\mathrm{crit}}}}$ occurs at $\alpha /\beta =2$ and $\lambda =0$, at which point $\beta _{{{}{\mathrm{crit}}}}=2$

**Fig. 7:**
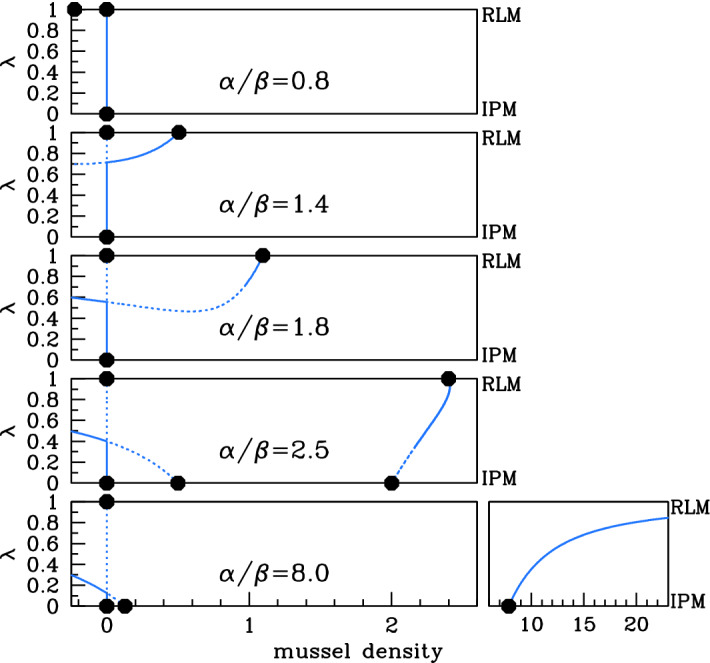
Steady states for $\xi <1$ and $\beta >2$. We plot mussel density *m* against the parameter $\lambda$ for the steady-state solutions of () when $\xi =0.15$ and $\beta =6$. Solid $\big /$ dashed lines indicate stable $\big /$ unstable steady states, calculated by numerical continuation using auto (Doedel 1981; Doedel et al. 1991, 2006), with $\lambda$ as continuation parameter. Dots indicate the steady states for the reduced losses ($\lambda =1$) and increased production ($\lambda =0$) models; these are $(a,m)=(\alpha ,0)$ plus (11), (12) and (13). The five different cases shown in the figure are separated by four critical values of $\alpha /\beta$: 1, $1/\lambda _{\text {{}min}}^{0}\approx 1.55$, 2, and $1/\xi \approx 6.67$; a full description is given in the main text. To improve clarity, the plot for $\alpha /\beta =8.0$ is shown in two parts with different horizontal magnifications. These plots should be compared with Fig. 3, which shows the corresponding plots when $\beta <2$: the steady states are the same but there are differences in stability for $\alpha /\beta =1.8$ and 2.5

Our method of numerical continuation gives the value of *m* at the (unique) positive steady state with positive Jacobian determinant at any point in the $(\alpha /\beta )$–$\lambda$ plane for which this steady state exists; these are the shaded regions in Fig. 5. Using this, we calculated $\beta _{{{}{\mathrm{crit}}}}$, and the results are shown as colour maps in Fig. 6. For both $\xi <1$ and $\xi >1$, the minimum value of $\beta _{{{}{\mathrm{crit}}}}$ is 2, occuring at $\lambda =0$ and $\alpha /\beta =2$; here, the value of $m=1$. Hence whenever $\beta <2$, ${{\mathcal {T}}}<0$ and stability depends only on the ratio $\alpha /\beta$. To illustrate the difference in steady-state stability that can occur for larger values of $\beta$, Fig. 7 shows the same bifurcation diagrams as in Fig. 3 but for $\beta =6$. The solution branch curves are the same, but for $\alpha /\beta =1.8$ and 2.5 the stability is clearly different.

Our objective is to study pattern formation in () as $\lambda$ varies between 0 and 1. Therefore, we require that there is a positive stable steady state for all values of $\lambda$, including the extreme case $\lambda =1$ (reduced losses model). This only occurs when $\xi <1$ and $2<\alpha /\beta <1/\xi$ (fourth panel in Fig. 3), and we restrict attention to parameter values satisfying these constraints, and also $\beta <2$.

## Spatial Pattern Formation

**Fig. 8:**
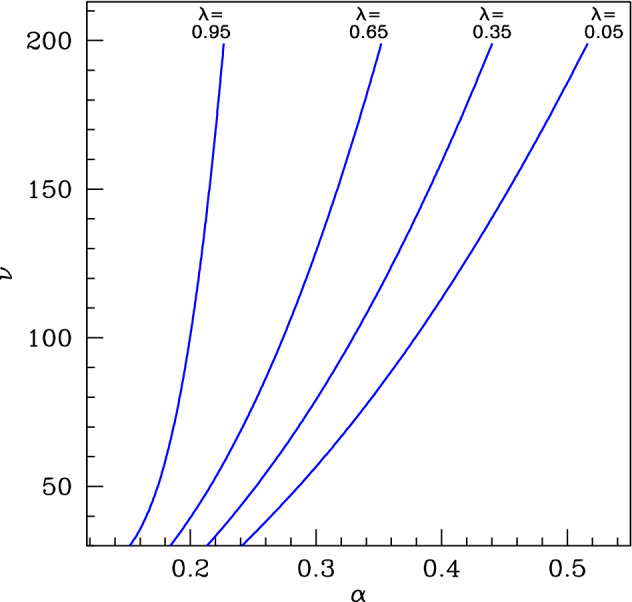
Pattern onset. We plot the patterning onset curves for () in the $\alpha$–$\nu$ plane for $\beta =0.1$ and $\xi =0.5$ fixed, for 4 values of the parameter $\lambda$. As $\alpha$ and $\nu$ are varied from lower right to upper left in the parameter plane, the locally stable positive steady state loses stability as the patterning onset curve is crossed. Note that in contrast to much of our work on steady states, the results in this and subsequent figures depend on the values of both $\alpha$ and $\beta$, rather than just their ratio. The curves were calculated by determining the dispersion relation as described in the main text and then solving numerically the condition for this dispersion relation to have a double root. The case $\lambda =0$ provides a starting point for this solution, since () then reduces to the Klausmeier model for which patterning conditions have been studied in detail in previous work (e.g. Sherratt 2005; Siteur et al. 2014; Sherratt 2015). From this starting point, we gradually increased $\lambda$, solving the double root conditions numerically using the solution at the previous value of $\lambda$ as an initial guess. Note that to enable direct comparison, the values of $\beta$ and $\xi$ used in this figure are the same as in Figs. 9, 10, 11, 12 and 13

When the parameters $\alpha$, $\beta$, $\xi$ and $\lambda$ are such that () has a positive steady state that is stable to spatially uniform perturbations, there is the possibility of pattern formation in the spatial model (), because the steady state can be destabilised by the advection and diffusion terms. This is sometimes known as “differential flow instability” (Rovinsky and Menzinger 1992). Conditions for patterning can be found via a standard procedure. We linearise equations () about the steady state $(a_s,m_s)$ and substitute $(a-a_s,m-m_s)=(a_0,m_0)\exp (ikx+\mu {}t)$. The requirement that the constants $a_0$ and $m_0$ are not both zero gives a dispersion relation, i.e. an equation for $\mu$ in terms of *k*. The steady state is stable if $\mathrm{Re}\,{\mu }<0$ for all real *k* and unstable if $\mathrm{Re}\,{\mu }>0$ for some real *k*. For a more detailed explanation, see Murray (2003), Perumpanani et al. (1995), Sherratt (2005), Siteur et al. (2014). Using this approach, we calculated the curves in the $\alpha$–$\nu$ plane on which the steady state loses stability, leading to patterns. This is illustrated in Fig. 8, which shows that the $\alpha$–$\nu$ region giving patterns shrinks as $\lambda$ is increased.

The curves plotted in Fig. 8 give an upper limit on the algal supply parameter $\alpha$ for spatial patterning, for given values of the other parameters. Intuitively, when $\alpha$ exceeds this upper limit the food supply to the mussels is large enough to maintain a spatially uniform mussel population. There is also a minimum value of $\alpha$ below which patterns do not occur, because the food supply is insufficient to maintain even a spatially patterned mussel population; this is discussed in more detail below. Between these two threshold values of $\alpha$ there are spatial patterns. Although the patterns have a constant shape, they are not stationary, but rather they move in the positive *x* direction (away from the shore) because of the directed movement of the algae (towards the shore). Mathematically, such movement is a standard feature of patterns in reaction–diffusion–advection equations (Rovinsky and Menzinger 1992; Perumpanani et al. 1995; Malchow 2000), and it is reflected by the imaginary part of the growth rate $\mu$ being nonzero. Intuitively, the model predicts that algal density is higher on the off-shore side of a mussel band compared to the on-shore side, because of consumption in the band. This causes a net growth of mussels on the off-shore side and a net loss on the on-shore side, and this causes a gradual net off-shore migration of the band (Song et al. 2017). Although such movement is intrinsic to patterned solutions of (), it is not observed in real mussel beds, presumably because the (deliberately) simplistic modelling omits some stabilising feature(s) of the real system—one possibility for this is the oscillatory nature of tidal flow, which has recently been incorporated into the reduced losses model () and which does lead to patterns without large-scale migration (Sherratt and Mackenzie 2016).

**Fig. 9:**
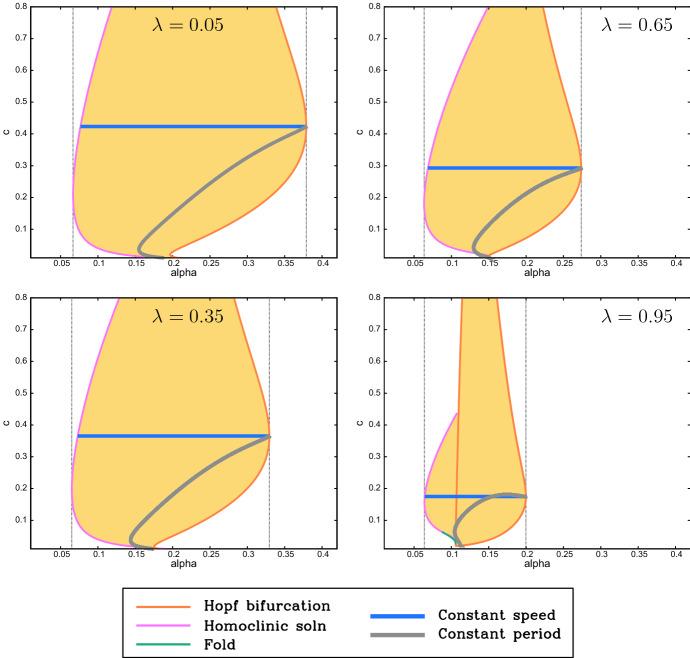
Pattern existence. We show examples of the regions (shaded) of the $\alpha$-migration speed plane in which patterns exist, for $\beta =0.1$, $\xi =0.5$ and $\nu =100$. The regions are bounded by loci of Hopf bifurcations, homoclinic solutions and solution branch folds in the travelling wave odes. The vertical dotted lines show the values of $\alpha$ between which there are pattern solutions of some speed. Thus, the right-hand vertical line corresponds to pattern onset: mathematically there is a Turing–Hopf bifurcation in () at this value of $\alpha$. We also show curves of constant migration speed and of constant wavelength (period) passing through the Turing–Hopf point. The various computations and plots were done using the software package wavetrain (Sherratt 2012). The shaded region includes patterns that are both stable and unstable as solutions of (). We include unstable patterns in the plots because they can be ecologically relevant in some situations (see, for example, Sherratt et al. 2009). Note that to enable direct comparison, the same parameters are used in Figs. 9, 10, 11, 12 and 13 . There is no special significance to the four values of $\lambda$ that we have chosen, and in particular our use of $\lambda =0.05$ and 0.95 rather than 0 and 1 is arbitrary

Being periodic solutions moving with constant shape and speed, the patterns are “periodic travelling waves”, and general theory for this type of solution implies that for a given value of the algal supply $\alpha$ (and of the other parameters) there is a family of patterns, with the migration speed and wavelength varying within the family (Sherratt and Smith 2008; Sherratt 2012; van der Stelt et al. 2013; Rademacher and Scheel 2007). A convenient way of illustrating the wave family is to plot the patterning region in the $\alpha$—speed plane, and some examples are shown in Fig. 9 with calculations done using the software package wavetrain (Sherratt 2012). In these plots, the patterning region is shaded; in keeping with Fig. 8 the region shrinks as $\lambda$ is increased. The right-hand boundary of the patterning region is a curve on which patterns with a particular speed are initiated: mathematically this is a locus of Hopf bifurcations in the travelling wave odes. The left-hand boundary is for the most part a curve along which the pattern wavelength becomes infinitely long: mathematically this is a locus of homoclinic solutions. For one of the cases shown in Fig. 9 ($\lambda =0.95$) a small part of the left-hand boundary consists instead of a locus of folds; this implies multiple pattern solutions in a small region of parameter space, but this has no practical significance for realistic parameters.

## Mussel Density in Spatial Patterns

**Fig. 10:**
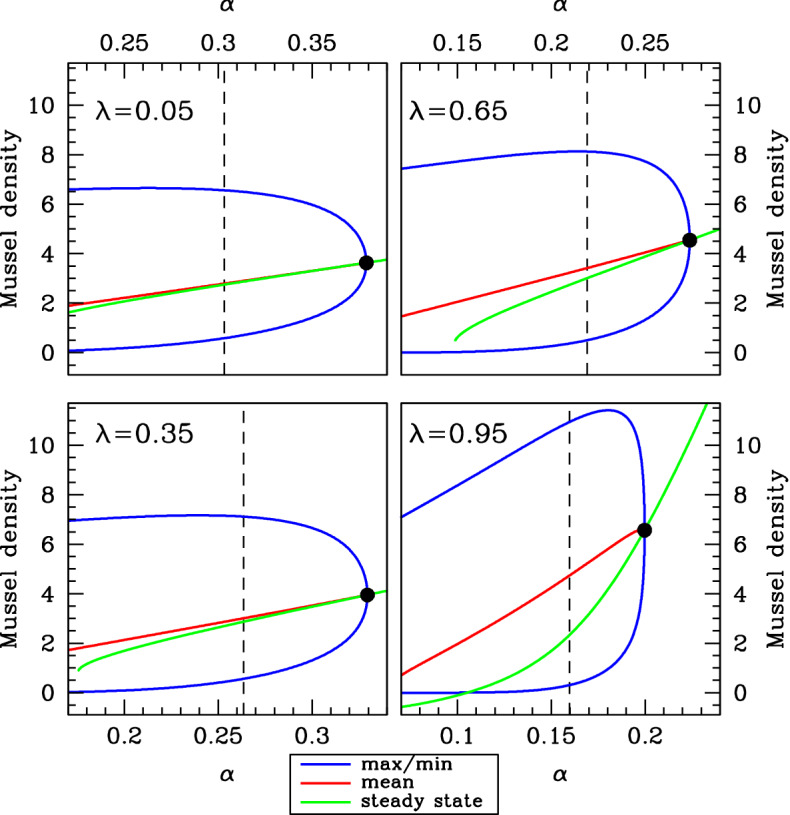
Density versus $\varvec{\alpha }$ at constant speed. We show comparisons of the average mussel density in spatial patterns with the steady-state mussel density, when the migration speed is held constant at its value at pattern onset (the Turing–Hopf bifurcation point, indicated by ). Using the software package wavetrain (Sherratt 2012), we tracked the form of the pattern along solution branches of () as $\alpha$ is varied with the other parameters fixed. We plot the maximum, minimum and mean (average) mussel density in the patterns and also the steady-state mussel density. The figure shows an increasing separation between the pattern and the steady-state solution as $\lambda$ is increased between 0 (increased production model) and 1 (decreased losses model). The dashed vertical lines indicate the values of $\alpha$ used in Fig.12. Note that to enable direct comparison, the same parameters are used in Figs. 9, 10, 11, 12 and 13

**Fig. 11:**
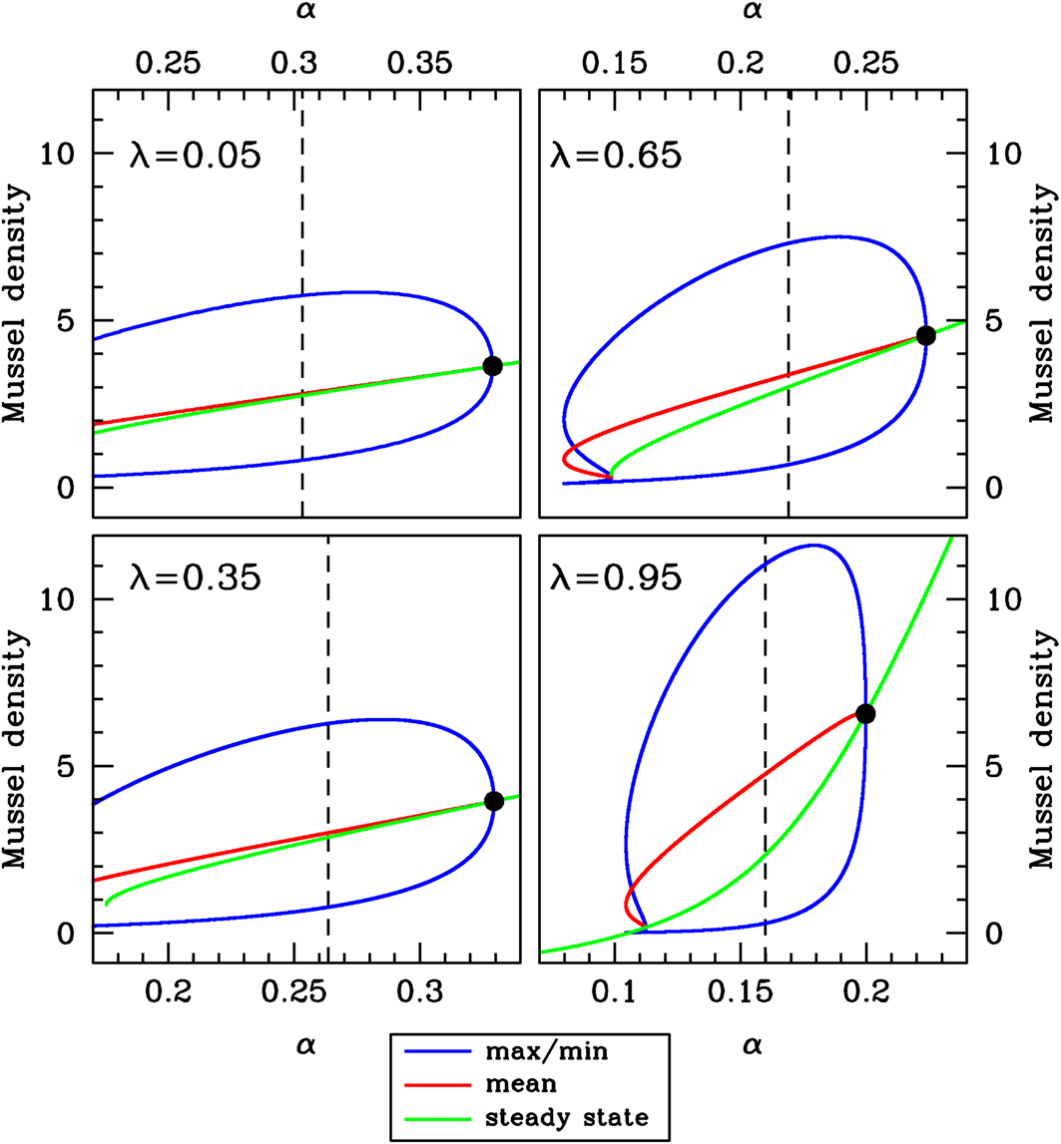
Density versus $\varvec{\alpha }$ at constant wavelength. We show comparisons of the average mussel density in spatial patterns with the steady-state mussel density, when the wavelength is held constant at its value at pattern onset (the Turing–Hopf bifurcation point, indicated by ). Using the software package wavetrain (Sherratt 2012), we tracked the form of the pattern along solution branches of () as $\alpha$ is varied with the other parameters fixed. We plot the maximum, minimum and mean (average) mussel density in the patterns and also the steady-state mussel density. As in Fig. 10, this figure shows an increasing separation between the pattern and the steady-state solution as $\lambda$ is increased between 0 (increased production model) and 1 (decreased losses model). The dashed vertical lines indicate the values of $\alpha$ used in Fig. 13. Note that to enable direct comparison, the same parameters are used in Figs. 9, 10, 11, 12 and 13

Our basic objective is to compare the average mussel density in spatial patterns with the density in the spatially uniform steady state from which the patterns have developed. Therefore, we tracked the form of the pattern along solution branches of () as $\alpha$ is varied with the other parameters fixed and compared this with the steady state for the same value of $\alpha$. Again we used the software package wavetrain (Sherratt 2012) for this calculation. Since there is a range of different patterned solutions for any value of $\alpha$ within the patterning range (see Fig. 9), it is necessary to impose some form of constraint in order to specify the solution branch to be followed. A number of simulation-based studies on semi-arid vegetation patterns (Sherratt 2013; Siteur et al. 2014; Siero et al. 2015) have found that as rainfall is varied slowly, the biomass within a vegetation pattern changes, but its wavelength remains the same—except for occasional large and abrupt changes in wavelength that occur when patterns of a given wavelength lose stability. Previous studies by one of us (Sherratt 2013, 2016) found a corresponding result in both the reduced losses model () and the increased production model () for mussel bed patterns. Therefore, in the present work we considered patterns of constant wavelength as $\alpha$ was decreased; we started our calculations at the pattern onset (Turing–Hopf bifurcation) point. However, our results do not depend critically on this assumption of constant wavelength, and to emphasise this we also considered patterns of constant speed (and therefore varying wavelength) as $\alpha$ was decreased. Both of the resulting solution branches are shown in Fig. 9. Figures 10 and 11 show typical results from these calculations. In both the constant speed and constant wavelength cases, there is an increasing separation between the pattern and steady-state solution branches as $\lambda$ is increased between 0 and 1. Recall that Liu et al. (2012) reported that the average mussel density in the spatial patterns was much greater than that in the steady state for the “reduced losses” model, while there was relatively little difference in the two densities for the “increased production” model. In our hybrid model (), there is a gradual transition from the increased production to the reduced losses feedback mechanism as $\lambda$ is increased at either constant speed or constant wavelength. Therefore, the results in Figs. 10 and 11 are consistent with the findings of Liu et al. (2012).

**Fig. 12:**
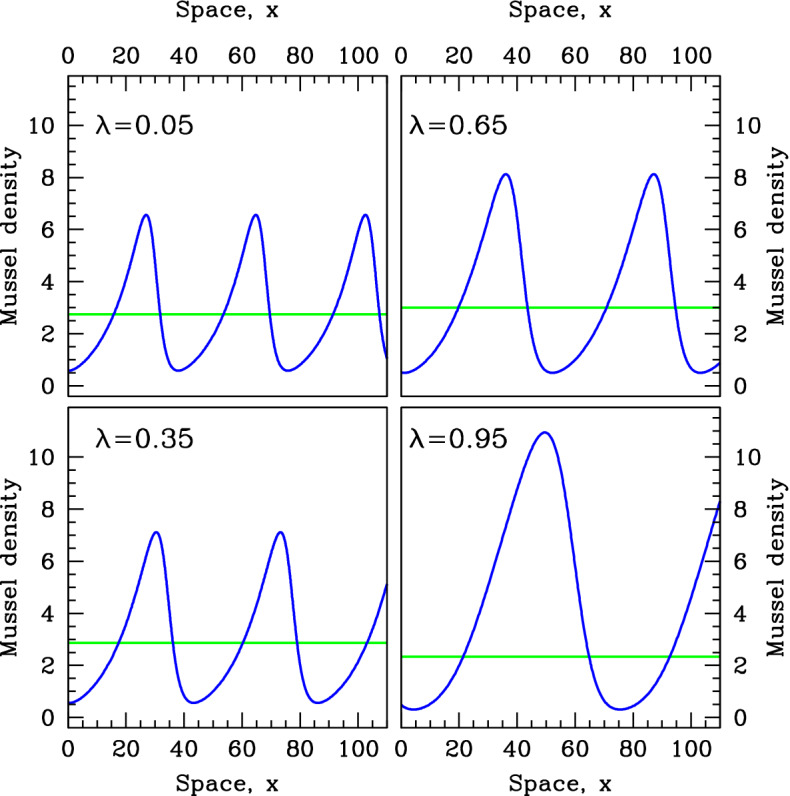
Patterns for the same speed as at the pattern onset point. We show examples of mussel density patterns given by model (). We plot patterns with the same migration speed as at the pattern onset (Turing–Hopf bifurcation) point, with the value of $\alpha$ set at 20% below that at the pattern onset point. These values of $\alpha$ are indicated by dashed vertical lines in Fig.10. In each panel, we also show the steady-state mussel density (horizontal line). The patterns were calculated by numerical continuation of the travelling wave odes starting at a Hopf bifurcation point, using the software package wavetrain (Sherratt 2012). Note that to enable direct comparison, the same parameters are used in Figs. 9, 10, 11, 12 and 13

**Fig. 13:**
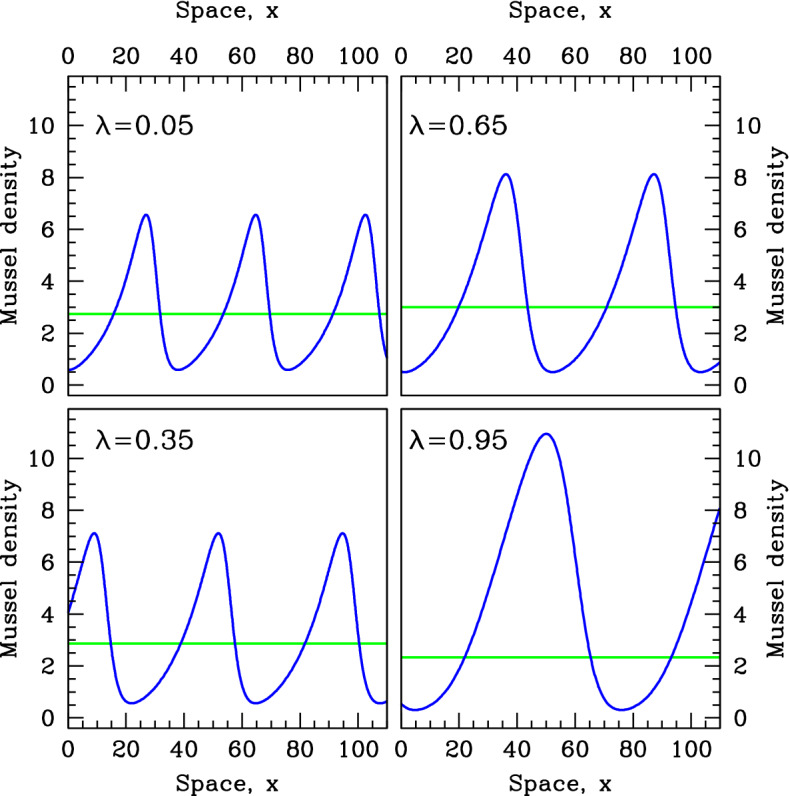
Patterns for the same wavelength as at the pattern-onset point. We show examples of mussel density patterns given by model (). We plot patterns with the wavelength as at the pattern-onset (Turing–Hopf bifurcation) point, with the value of $\alpha$ set at 20% below that at the pattern onset point. These values of $\alpha$ are indicated by dashed vertical lines in Fig. 11. In each panel, we also show the steady-state mussel density (horizontal line). The patterns were calculated by numerical continuation of the travelling wave odes starting at a Hopf bifurcation point, using the software package wavetrain (Sherratt 2012). Note that to enable direct comparison, the same parameters are used in Figs. 9, 10, 11, 12 and 13

The vertical dashed lines in Figs. 10 and 11 are at values of $\alpha$ that are 20% below the pattern-onset values. In Figs. 12 and 13, we show the corresponding patterns, plotting mussel density against space. The steady-state mussel density is also indicated in these figures. This shows that in contrast to the increased production mechanism, feedback of decreased losses type ($\lambda$ near 1) causes high mussel densities in the peaks of the patterns; it is this that leads to the high level of mean mussel density, compared to the steady state.

**Fig. 14:**
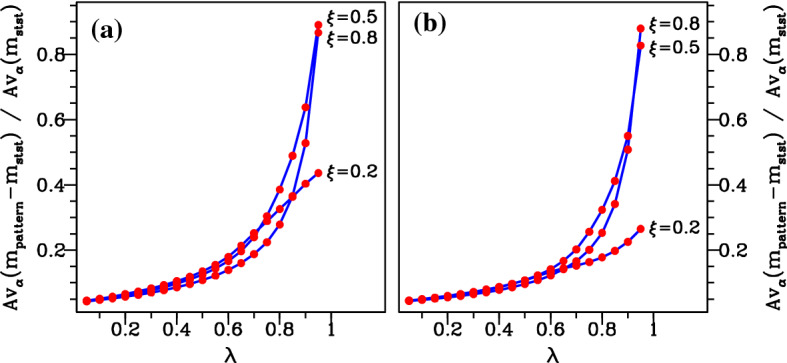
Quantitative details of the comparison between the average mussel density in spatial patterns and the steady-state mussel density. In (**a**, **b**) the migration speed and wavelength, respectively, are held constant at their value at pattern onset (the Turing–Hopf bifurcation point); examples of the two corresponding solution branches are shown in Fig. 9. For each pair of $\lambda$ and $\xi$ values, we used the software package wavetrain (Sherratt 2012) to track the form of the pattern along these solution branches as $\alpha$ is varied with the other parameters fixed. We then calculated the average over $\alpha$ of the difference between the mean mussel density and the steady-state mussel density and divided this by the average of the steady-state mussel density. This gives a single number comparing the mussel density in spatial patterns and in the steady state, and we plot this as a function of $\lambda$ for $\xi =0.2$, 0.5 and 0.8. The plots show an increasing separation between the pattern and steady-state solution branches as $\lambda$ is increased between 0 (increased production model) and 1 (decreased losses model). However, there is no clear trend in the way in which the difference in mussel densities varies with the parameter $\xi$. The other parameters are $\beta =0.1$ and $\nu =100$

To quantify the difference between the pattern and steady-state mussel densities, we calculated a single numerical measure of this difference. The starting point for our calculation is the variation with $\alpha$ of the mean mussel density in patterns and of the steady-state mussel density; typical examples of this are illustrated in Figs. 10 and 11. We then calculated the average over $\alpha$ of the difference between the two densities and divided this by the average of the steady-state density. We restricted our averaging to values of $\alpha$ for which the pattern and the steady state both exist, and for which the steady state is positive. In some cases, there is a fold in the pattern solution branch (for example the $\lambda =0.95$ case in Fig. 11), and then, we restricted attention to the part of the solution branch between the fold and the pattern onset (Turing–Hopf bifurcation) point. This prevents the results from being skewed by patterns with very low densities that are (typically) unstable as solutions of (). We repeated this procedure for a sequence of values of $\alpha$, and for three values of $\xi$, for both the constant speed and constant wavelength solution branches. The results are shown in Fig. 14. This figure illustrates the broad generality of two conclusions. First, the (average) mussel density in the patterns is greater than that in the steady state—this follows from all of the differences plotted in Fig. 14 being positive. And secondly there is a gradual increase in the average difference in mussel density as $\lambda$ is increased from 0 to 1, i.e. as the feedback mechanism gradually shifts from increased production to decreased losses.

## Recovery Time

**Fig. 15:**
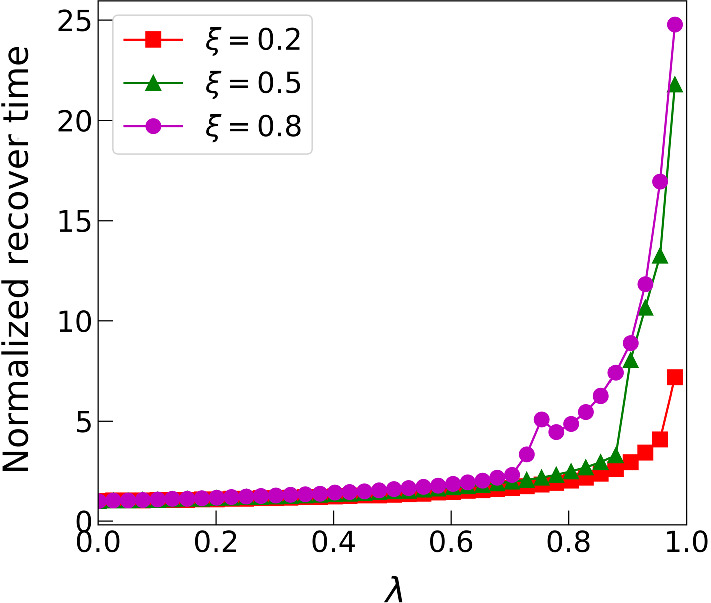
Recovery time versus $\varvec{\lambda }$. Quantitative comparison of recovery time from directional numerical simulation on model (). A spatial pattern is generated in model (); then, a single pattern period was extracted and a perturbed version of this was used as an initial condition on a spatial domain of length equal to one wavelength, with periodic boundary conditions. The perturbation consisted of a removal of 20% of the total biomass, varying randomly in space. The “recovery time” is a measure of the time taken for the solution to return to the patterned state; it is defined as the time by which solution is within 99% of the pre-perturbation pattern. We found that the details of the perturbation had a negligible effect on the recovery time. There is a clear trend for this recovery time to increase with the parameter $\lambda$, that is as the feedback mechanism gradually shifts from increased production to decreased losses. The parameter values are $\alpha =0.2$, $\beta =0.1$, $\nu =100$

Previous modelling predicts that self-organisation into spatial patterns significantly increases the resilience of mussel beds to disturbances, relative to the resilience of spatially uniform mussel populations (van de Koppel et al. 2005; Liu et al. 2012). Moreover, in their study comparing models based on the reduced losses and increased production mechanisms, Liu et al. (2012) reported that this increased resilience occurs to a greater extent in the reduced losses model than in the model based on increased production. However, it is again unclear to what extent this difference depends on the formulation of the models and the parameter values, rather than being due to the alternative underlying patterning mechanisms. To clarify this, we investigated resilience in our hybrid model (), by removing a fixed proportion of the total mussel population from a patterned solution, via a spatially varying perturbation, and monitoring the time taken to return to the original pattern. A typical result is shown in Fig. 15, confirming that the recovery time increases gradually as one varies the parameter $\lambda$ from $\lambda =0$ (increased production) to $\lambda =1$ (reduced losses). This shows that the difference in resilience can indeed be attributed to the underlying pattern mechanism, rather than being a function of parameter values or other model details.

## Discussion

The concept of self-organisation has been particularly successful in providing a framework for the emergence of spatial patterns in a wide range of systems. Despite its generality, a wide array of possible self-organisation models have now emerged for an equally large array of example ecosystems, and attempts for unification have been limited. This limits our understanding of the functional differences between self-organised ecosystems in a comprehensive way. Here we analysed a generalised model of pattern formation in ecosystems using a model that combines two general mechanisms for pattern formation: “increased production” and “reduced losses”. We showed that both mechanisms predict Turing-type regular patterns to develop, but that there are clear differences between the two models.

In reality, the reduced losses and increased production mechanisms are not alternatives—both will apply to some extent, and they act in concert to generate spatial patterns. Our hybrid model () provides a means of including both mechanisms, with a single new parameter, and our detailed study of pattern generation provides a comprehensive framework for understanding the solutions of this model. A key take-home message of our work concerns sensitivity to variations in the balance between the reduced losses and increased production mechanisms. Figures 14 and 15 show that a change in this balance has a much greater effect in a system that is dominated by the reduced losses mechanism than for a system dominated by the increased production mechanism. This highlights that when the reduced losses mechanism is dominant, it is particularly important to investigate the possibility and extent of additional feedback mechanisms.

Our work has been set in the specific context of spatially patterned mussels beds. However, one or other of the “decreased losses” and “increased production” mechanisms lie at the heart of many other patterned ecosystems. Vegetation patterns are a characteristic feature of semi-arid ecosystems, consisting of alternating patches of vegetation and bare ground (Bastiaansen et al. 2018; Gandhi et al. 2019). It is widely accepted that a mechanism of increased production type plays a key role in the generation of these patterns; specifically, higher vegetation levels increase the rate at which rain water infiltrates into the soil, leading to higher plant growth rates (Klausmeier 1999; Rietkerk et al. 2002; Meron 2012). Indeed, as we have already remarked, the $\lambda =0$ limit of () gives the widely used Klausmeier model for vegetation patterning (Klausmeier 1999; Sherratt and Lord 2007; van der Stelt et al. 2013). Another ecosystem in which a mechanism of “increased production” type drives pattern formation is the sequence of ridges and hollows found in many peat bogs (Morris et al. 2011). In fact, two different mechanisms combine to generate these patterns, with each mechanism being of “increased production” type. The elevated ridges are drier than neighbouring hollows, which causes an increased production rate of peat and an increase in the thickness of the surface acrotelm layer. This further amplifies the height difference between the ridges and hollows. In addition, the higher evapotranspiration rates by the vascular plants on the ridges lead to increased nutrient accumulation, which generates faster plant growth and thus a thicker acrotelm layer. Mathematical models based on these mechanisms confirm pattern formation (Morris et al. 2011; Eppinga et al. 2009) and have been verified by comparison with field data (Eppinga et al. 2008).

An example of an ecosystem in which pattern formation is driven by a mechanism of “reduced losses” type is provided by patterns of hummocks and hollows on intertidal mudflats. Diatoms accumulate on the top of hummocks, forming a biofilm that is strengthened by extracellular secretions, and this promotes sedimentation; in the hollows, water accumulation inhibits the corresponding processes. This was first proposed as a pattern generation mechanism by Rietkerk and van de Koppel (2008) and has subsequently been tested via both modelling and field data (Weerman et al. 2010).

The occurrence of the two alternative mechanisms in a wide range of ecosystems highlights the importance of understanding the similarities and differences in the patterns that they generate. In particular, the combination of modelling studies and field work has the potential to distinguish the two mechanisms, and our results open up a number of different avenues for such future cross-disciplinary research.

## Appendix

In Fig. 1, we show a comparison between two- and three-equation models for the increased production mechanism. As a prelude to these simulations, we derived dimensionless versions of the models that can easily be compared. Rescalings (6) do not extend to the three-equation model () in a natural way; therefore, we use instead the rescalings proposed by Liu et al. (2012):

$$\begin{aligned} \check{t}= & {} \widehat{E}\,\widehat{t}\qquad \check{x}=\left( \frac{\widehat{E}}{\widehat{D}_m}\right) ^{1/2}\widehat{x}\qquad \check{a}=\left( \frac{\widehat{E}}{\widehat{F}\widehat{A}}\right) \widehat{a}\qquad \check{m}=\left( \frac{\widehat{P}}{\widehat{E}\widehat{S}_0}\right) \widehat{m}\qquad \check{s}=\widehat{s}\big /\widehat{S}_0 \\ \check{\alpha }= & {} \widehat{F}\big /\widehat{E}\qquad \check{\beta }=\widehat{B}\widehat{S}_0\big /\widehat{P}\qquad \check{\nu }=\left( \widehat{E}\widehat{D}_m\right) ^{-1/2}\widehat{\nu }\qquad \check{\eta }=\widehat{\eta } \\ \check{D}= & {} \widehat{D}_s\big /\widehat{D}_m\qquad \check{\theta }=\widehat{Q}\big /\widehat{E}\qquad \check{\delta }=\widehat{C}\widehat{F}\widehat{A}\big /\widehat{E}^2 . \end{aligned}$$

This transforms () to the system

A.1a $$\begin{aligned} \partial \check{a}/\partial \check{t}= & {} 1-\check{\alpha }\check{a} -\check{\beta }\check{a}\check{m} (\check{s}+\check{\eta })/(\check{s}+1) +\check{\nu }\,\partial \check{a}/\partial \check{x} \end{aligned}$$

A.1b $$\begin{aligned} \partial \check{m}/\partial \check{t}= & {} \check{\delta }\check{a}\check{m} (\check{s}+\check{\eta })/(\check{s}+1)- \check{m} + \partial ^2\check{m}/\partial \check{x}^2 \end{aligned}$$

A.1c $$\begin{aligned} \partial \check{s}/\partial \check{t}= & {} \check{m}-\check{\theta }\check{s} +\check{D}\,\partial ^2\check{s}/\partial \check{x}^2. \end{aligned}$$

Applying the same rescalings to () gives

A.2a $$\begin{aligned} \partial \check{a}/\partial \check{t}= & {} 1-\check{\alpha }\check{a} -\left( \check{\beta }\big /\check{\theta }\right) \check{a}\check{m}^2 +\check{\nu }\,\partial \check{a}/\partial \check{x} \end{aligned}$$

A.2b $$\begin{aligned} \partial \check{m}/\partial \check{t}= & {} \left( \check{\delta }\big /\check{\theta }\right) \check{a}\check{m}^2 -\check{m} + \partial ^2\check{m}/\partial \check{x}^2. \end{aligned}$$

In Fig. 1 we compare solutions of () and () for parameter values taken from Liu et al. (2012). The uniform steady state $(\check{a}_s,\check{m}_s,\check{s}_s)$, which is used in the initial conditions for the simulations in Fig. 1, is given by

$$\begin{aligned} \check{s}_s= & {} \frac{\check{\delta }-\check{\alpha }- \check{\beta }\check{\theta }\check{\eta }+\sqrt{\left( \check{\delta }-\check{\alpha }- \check{\beta }\check{\theta }\check{\eta }\right) ^2-4\check{\beta }\check{\theta } \left( \check{\alpha }-\check{\delta }\check{\eta }\right) }}{2\check{\beta }\check{\theta }}\\ \check{m}_s= & {} \check{\theta }\check{s}_s\\ \check{a}_s= & {} \frac{1+\check{s}_s}{\check{\delta }\left( \check{s}_s+\check{\eta }\right) } \end{aligned}$$

(see Liu et al. 2012 for details).

One notable feature of Fig. 1 is that in the solution of the three-equation model, the patterns for $\check{m}$ and $\check{s}$ are approximately in phase. Motivation for this small phase difference comes from linear stability analysis of the spatially uniform steady state from which patterns bifurcate. Linearising () about this steady state and substituting $(\check{a}-\check{a}_s,\check{m}-\check{m}_s,\check{s}-\check{s}_s)= (\check{a}_0,\check{m}_0,\check{s}_0)\exp (ik\check{x}+\mu \check{t})$ gives

$$\begin{aligned} \left( \lambda +\check{\theta }+\check{D}k^2\right) \check{s}_0 = \check{m}_0. \end{aligned}$$

Therefore, the phase difference between the $\check{m}$ and $\check{s}$ patterns at the Turing–Hopf bifurcation point is

$$\begin{aligned} \arg \left[ \left( \mathrm{Re}\,\lambda +\check{\theta }+\check{D}k^2\right) +i\,\mathrm{Im}\,\lambda \right] . \end{aligned}$$

Since $\left| \mathrm{Im}\,\lambda /k\right|$ is the pattern migration speed, which is small, the phase difference is also small. There is no *a priori* reason for the phase difference not to increase, potentially significantly, as the pattern amplitude increases, but our numerical simulations suggest that this does not happen.

## Footnotes

1 This statement is based on evidence from our numerical continuation study, but is not proven. In particular, there could in principle be a closed branch of spatially uniform steady states that exists for a range of values of $\lambda$ in (0, 1) but does not extend to either $\lambda =0$ or $\lambda =1$. However, we have not found any evidence of such a closed branch.

2 Denoting by $m_s$ the steady-state value for *m*, ${{\mathcal {F}}}_2=1+\lambda {}m_s+(1-\lambda )m_s^2$ and $\mathcal{F}_1=\bigl [ (1-\lambda )(1+\xi {}m_s)(1+(1-\lambda )\xi {}m_s)+ \bigl (\lambda +(1-\lambda ){}m_s\bigr )\lambda \xi {} \bigr ]m_s\big / \bigl [(\lambda +(1-\lambda ){}m_s){}(1+\xi {}m_s)^2\bigr ] .$
